# The artificial pancreas: two alternative approaches to achieve a fully closed-loop system with optimal glucose control

**DOI:** 10.1007/s40618-023-02193-2

**Published:** 2023-09-15

**Authors:** M. K. Åm, I. A. Teigen, M. Riaz, A. L. Fougner, S. C. Christiansen, S. M. Carlsen

**Affiliations:** 1 Department of Clinical and Molecular Medicine, Faculty of Medicine and Health Sciences, Norwegian University of Science and Technology, Postboks 8900, 7491 Trondheim, Norway; 2 Cancer Clinic, St. Olav’s Hospital, Trondheim University Hospital, Trondheim, Norway; 3 Department of Engineering Cybernetics, Faculty of Information Technology and Electrical Engineering, Norwegian University of Science and Technology, Trondheim, Norway; 4 Department of Endocrinology, St. Olav’s Hospital, Trondheim University Hospital, Trondheim, Norway

**Keywords:** Diabetes mellitus, Type 1, Blood glucose, Glucagon, Insulin infusion system

## Abstract

**Introduction:**

Diabetes mellitus type 1 is a chronic disease that implies mandatory external insulin delivery. The patients must monitor their blood glucose levels and administer appropriate insulin boluses to keep their blood glucose within the desired range. It requires a lot of time and endeavour, and many patients struggle with suboptimal glucose control despite all their efforts.

**Materials and methods:**

This narrative review combines existing knowledge with new discoveries from animal experiments.

**Discussion:**

In the last decade, artificial pancreas (AP) devices have been developed to improve glucose control and relieve patients of the constant burden of managing their disease. However, a feasible and fully automated AP is yet to be developed. The main challenges preventing the development of a true, subcutaneous (SC) AP system are the slow dynamics of SC glucose sensing and particularly the delay in effect on glucose levels after SC insulin infusions. We have previously published studies on using the intraperitoneal space for an AP; however, we further propose a novel and potentially disruptive way to utilize the vasodilative properties of glucagon in SC AP systems.

**Conclusion:**

This narrative review presents two lesser-explored viable solutions for AP systems and discusses the potential for improvement toward a fully automated system: A) using the intraperitoneal approach for more rapid insulin absorption, and B) besides using glucagon to treat and prevent hypoglycemia, also administering micro-boluses of glucagon to increase the local SC blood flow, thereby accelerating SC insulin absorption and SC glucose sensor site dynamics.

## Introduction

Diabetes mellitus type 1 (DM1) is a chronic metabolic disease caused by the autoimmune destruction of the insulin-producing β-cells in the pancreas, leaving the patients dependent on an external supply of insulin. The treatment of DM1 has significantly evolved, since insulin was discovered and became available to patients hundred years ago. However, the core of treatment remains the same, i.e., controlling blood glucose levels with insulin replacement therapy. Different insulin formulations and analogues have been developed over the years, leading to a significant improvement in the management of DM1. One major improvement is the development of fast-acting insulin analogues that more efficiently reduce postprandial glucose excursions, and new, even faster-acting formulations continue to reach the market [1, 2]. Another substantial improvement in diabetes treatment in the last decades includes the development of continuous subcutaneous (SC) insulin infusion pumps (insulin pumps) and sensors for SC continuous glucose monitoring (CGM). In 2021, 35% of Norwegian patients with DM1 were treated with insulin pumps, and 80% of patients used CGMs [3]. Despite the increased use of diabetes technology, the average glucose control evaluated by glycated haemoglobin A1c (HbA1c) levels has not improved as wanted compared to conventional treatment [4]. However, a meta-analysis showed a greater potential for improved glucose control with AP systems compared with individual insulin pumps and CGM [5]. It indicates that self-regulation of glucose levels is still challenging; many patients continue to experience frequent and long-lasting hyperglycemia, severe hypoglycaemia, subsequent diabetic organ complications, reduced life expectancy, and reduced quality of life [6, 7]. The struggle to achieve strict glucose control also increases the risk of hypoglycemia and premature death [8, 9].

The last decade has seen further development in DM1 treatment technology as automated insulin delivery systems, i.e., artificial pancreas (AP) systems, have reached the market. An AP has become possible due to the availability of SC CGM systems. Currently, only single-hormone hybrid AP systems (often called hybrid closed loop, HCL) are commercially available. The term “hybrid” reflects that these systems still require patients to inform the device of the amount of carbohydrates they intend to consume, so the AP can calculate the proper amount of insulin to be given with the meal. Thus, DM1 patients using hybrid AP systems still must keep a daily focus on their disease management. For some users, the frequent alarms and need for interventions are strenuous and lead to stress, insomnia, fatigue, and discontinuation of AP use due to “alarm fatigue” [10].

The most frequently used parameter to evaluate the performance of AP systems is the percentage of time spent within the normoglycemic range, i.e., time with blood glucose concentrations between 3.9 mmol/l and 10.0 mmol/l (time in range, TIR). It should be noticed that the desired glucose range for patients with DM1 is wider than the glucose range experienced by healthy people. Different studies yield varying results, but studies on single-hormone hybrid AP systems in a home setting report TIR in the 65–80% range [11–19]. AP systems perform significantly better during night-time than daytime, probably because meals and physical activity are the two most challenging situations to handle for an AP.

Presently, all commercially available AP systems and most AP research focus on the single-hormone double SC approach, i.e., CGM and insulin delivery in SC tissue. Unfortunately, this approach struggles with inherent slow dynamics in CGM and in the absorption and the glucose-lowering effect of meal-time insulin boluses. The limitations of a single-hormone double SC AP pancreas were comprehensively discussed in a review paper from 2019 [20]. The situation has not changed substantially since then, although bihormonal double SC AP systems have been further explored, and new fast-acting insulin analogues have emerged on the market. Adding glucagon to the treatment algorithm allows for more aggressive insulin treatment, as glucagon is used to counteract insulin-induced hypoglycemia or predicted hypoglycemia in the near future. Thereby, the bihormonal SC approach improves glucose control and increases TIR. With the bihormonal approach, at best, TIR has been shown to increase to 80–85% in short-term studies (≤ 14 days) [21–24]. However, the ideal AP with 100% TIR and no need for meal announcement, which would carry the promise of totally eradicating the long-term adverse effects of having DM1, is still far away.

There are several ways to compensate for the slow dynamics mentioned above, either by directly addressing the physiological causes of the slow dynamics in glucose sensing and insulin absorption, or by adding components to the AP that detect meals/physical activity earlier. The Artificial Pancreas Trondheim research group (APT) has proposed using the double intraperitoneal (IP) approach, by measuring glucose and delivering insulin in the IP space. This approach was reviewed in 2016 [25]. In short, the main reason for an IP approach is the faster dynamics in glucose sensing and insulin absorption (and its glucose-lowering effect) compared to the SC approach. Later, in several animal studies, we explored the IP approach for both insulin and glucagon [26–29]. Recently, we also discovered a previously unrecognized local effect of micro-boluses of glucagon on cutaneous blood flow [30]. This paper details the factors associated with the slow dynamics of AP systems and outlines viable solutions and a roadmap toward achieving a fully closed-loop AP with superior glucose control.

## Possible approaches for fully automated AP systems

The two major physiological limitations in the AP system are the slow dynamics in glucose sensing and particularly the delayed insulin absorption and effect on glucose metabolism that emerges too late to prevent excessive postprandial glucose excursions. In this narrative review, we discuss two viable solutions for overcoming these difficulties; (A) the IP approach with IP insulin and SC CGM and glucagon, and (B) the SC approach with SC insulin, CGM and glucagon, which uses micro-boluses of glucagon as an SC vasodilator to enhance insulin absorption.

### The intraperitoneal approach

From a physiologic point of view, an IP approach seems ideal for an artificial pancreas. IP insulin delivery results in faster insulin effect, stricter glucose control, and fewer hypoglycaemic events [31, 32]. We and others have modelled that by going IP, the delays in the closed loop are minimized to the degree that will make a fully automated AP with nearly 100% TIR possible [33, 34]. Insulin is absorbed significantly faster and has an earlier onset of effect when delivered IP compared to SC [31]. It is absorbed into the vessels in the peritoneal lining, transported via the portal vein, and reaches the liver at high concentrations before entering systemic circulation. We have shown in anesthetized pigs (35–40 kg) that most of the insulin after small and medium boluses (2 and 5 U) is removed by the first-pass effect in the liver and can only be detected in the systemic circulation when the dose is doubled (10 U), and the liver is saturated with insulin [29]. Another theoretical advantage of IP insulin delivery is that the core temperature, contrary to the temperature in SC tissue, is stable. This means that unlike SC blood flow, which is essential to body temperature regulation and can vary by a factor of eight, the blood flow below the peritoneal lining varies less, which may translate into a more predictable speed of insulin absorption and, thus, a more predictive effect on glucose levels, which would be an obvious advantage in any AP.

However, the overall benefit of using the IP space for glucose sensing and glucagon delivery is questionable. Experiments on pigs with amperometric sensors have conveyed conflicting results, indicating either significantly faster or minimally faster glucose kinetics in the IP space compared to the SC tissue [35, 36]. On the other hand, an experimental sensor in development for intravascular CGM achieved near real-time CGM in the IP space, i.e., the sensor detected a blood glucose elevation with a time delay of only 0–30 s and time constants of only 0.5–10 min [37] which is significantly less than what you experience in the SC tissue. The superior performance of the latter sensor probably was because it measured the glucose at the surface of the peritoneal lining in contrast to the amperometric sensors, which measured glucose in the peritoneal fluid [38]. As far as we know, no IP CGM solution is yet available or in the immediate pipeline. Our research group is working on establishing IP CGM with new innovative methods [39, 40], and other sensing technologies under development might also be used IP [41]. However, the long-term use of an IP CGM will at best be challenging with biofouling of the sensor resulting in declining sensor performance and non-reliable CGM values.

For glucagon delivery, we have shown in both rats and pigs that the pharmacodynamics of glucagon injected subcutaneously are comparable to glucagon given intraperitoneally [27, 28]. Theoretically, the probability of adverse effects is reduced by IP administration of glucagon, since smaller doses are sufficient and less glucagon reaches systemic circulation. However, the possible overall benefit of IP delivery of glucagon compared with SC delivery in an AP setting remains to be established.

Regardless of the number of IP components, the major challenge with the IP approach for an AP is its invasive nature. There are concerns regarding the possibility of adverse effects, such as infections, the complexity of the implantation process, and the uncertainty of how patients with DM1 will accept such an AP. The AP device itself is also complex, regardless of the use of external or fully implanted pumps. Catheter obstructions caused by the formation of insulin amyloid aggregates and foreign body reactions are also a barrier to using this solution in a broader population of patients. A bihormonal IP AP is only feasible with the use of stable liquid glucagon formulations. Two new glucagon analogues for treating hypoglycemia with 4 weeks of durability at room temperature are approved by FDA. However, in the case of an implanted IP AP, the stability might be reduced, because the storage temperature will be close to 37 degrees centigrade. However, for an external based IP AP, this issue will be the same as in an SC AP.

**Fig. 1:**
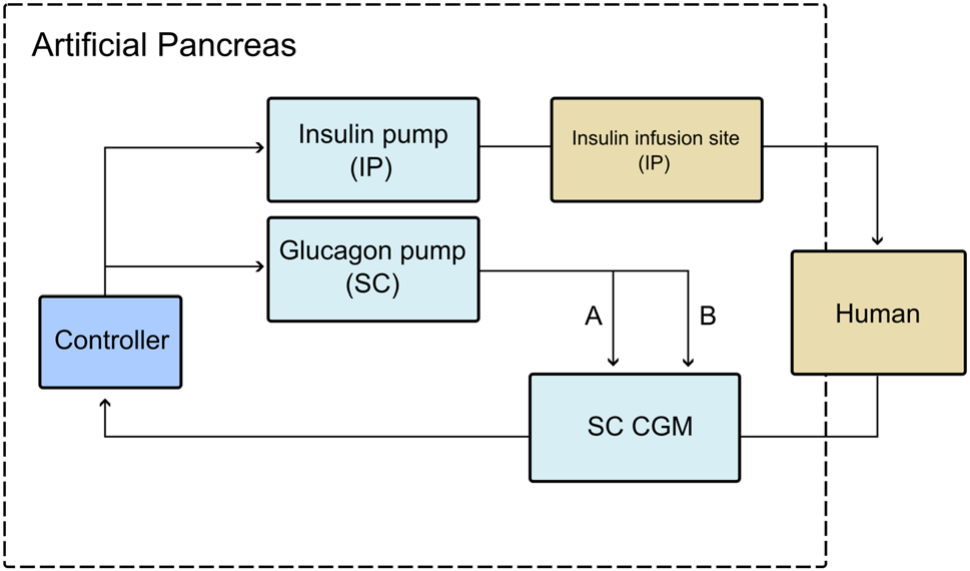
Illustration of an intraperitoneal (IP) artificial pancreas with possible use of glucagon to achieve a fully closed-loop system; **A** glucagon is used to prevent or treat hypoglycemia, **B** glucagon micro-boluses are used to enhance continuous glucose monitoring (CGM) performance by increasing local SC blood flow. If glucagon is not used to enhance CGM performance, it will be delivered at another SC site than CGM

As mentioned above, there is probably little to be gained in terms of improved glucose control by utilizing the IP space for glucagon delivery or CGM with the current technology. However, an approach with IP insulin, SC CGM, and SC glucagon could be possible within a reasonable timeline (Fig. 1). SC CGM systems are readily available; a bihormonal pump or two separate pumps can be used, and IP insulin can be delivered through the abdominal port from Roche (DiaPort). This solution should achieve close to as good glucose control as the triple IP approach, even if micro-boluses of glucagon at the site of the SC CGM sensor (to be discussed below) only marginally improve glucose sensing while being technically much less complex. This approach can also be used with separate SC sites for CGM and glucagon delivery and IP insulin delivery taking advantage of the faster IP insulin effect [31], the equal SC versus IP glucagon effect and acknowledging the at least 6–7 min delay in SC CGM adds little to de combined delay in an SC AP compared to the substantially delayed insulin effect.

### The subcutaneous approach, exploiting glucagon as a vasodilator

Glucagon is traditionally added to the AP system to antagonize the glucose-lowering effect of insulin, thereby allowing more aggressive insulin treatment. Bihormonal double SC AP has been studied in a few short-term studies in patients with DM1 and after pancreatectomy, including outpatient trials [21–24, 42]. Results show a moderate improvement with TIR between 80 and 85% in studies lasting ≤ 14 days [21–24]. The time in hypoglycemia (TIH, glucose < 3.9 mmol/L) is significantly reduced, which is a substantial improvement for some DM1 patients [21–24]. The study by Blauw et al. is of special note, which is the first to study the effect of a true AP with no meal announcements by users [21]. This short-term (14 days) study presents a median TIR of 86.6% and illustrates that an SC bihormonal true AP may be possible, although measures to compensate for postprandial hyperglycemia are still warranted. However, the study results should be cautiously interpreted, as patients may have been particularly dedicated and cautious during this limited study period. Long-term studies in non-selected DM1 patients might show quite different results as the initial positive effect of many interventions in patients with DM1 tend to decrease after 6–12 months.

The slowness in SC insulin absorption and effect on blood glucose levels represents the major limitation of an SC AP and is the main reason for the bihormonal SC AP’s inability to achieve perfect glucose control, as discussed previously in this paper. Means to accelerate the absorption of insulin will enable an SC AP to regulate postprandial glucose elevations more efficiently. The insulin absorption rate from SC tissue depends on several factors, such as type of insulin formulation, concentration, added excipients, skin temperature, local blood flow, injection site, injection technique, lipohypertrophy, obesity, and comorbidities associated with diabetes [43]. In addition, there is substantial intraindividual and interindividual variability in SC insulin pharmacokinetics and pharmacodynamics. Prior research proves that manipulating SC blood flow (tissue perfusion) using heat or local massage can significantly alter the rate and amount of insulin absorption [44]. Recently, Eli Lilly has developed an ultra-rapid insulin formulation (Lyumjev^®^) by adding the vasodilating drug treprostinil to the fast-acting insulin analogue insulin lispro. Treprostinil speeds up the local absorption, resulting in a faster onset of effect, and thus reduced postprandial glucose excursions [2]. In this insulin formulation, the concentration of treprostinil is low, only 10 ng/unit insulin or 1 µg/ml; however, it achieves significantly faster SC absorption and an earlier onset of glucose-lowering effect than conventional fast-acting insulins [45]. These observations illustrate how local vasodilation affects the rate of insulin absorption. Even though the effect of slow dynamics in SC CGM is less pronounced than for insulin absorption, increasing local SC blood flow might also enhance the CGM performance and, consequently, the performance of an SC AP system. This idea of enhancing CGM performance has only briefly been investigated [46], but it is commonly experienced by DM1 patients that changes in skin temperature and episodes of mechanical pressure affect glucose readings due to the change in the local skin blood flow [47, 48]. To achieve optimal glucose control by improving the dynamics of the AP system mentioned above, we will further in the text debate the possibility of a bihormonal AP that additionally employs glucagon micro-boluses as a vasodilator to achieve a fully closed loop.

The SC pharmacokinetics of glucagon is significantly faster that the SC pharmacokinetics of insulin. We believe that can be explained by glucagon’s vasodilating properties. Aside from being used as a drug to reduce hypoglycemia, glucagon is also used to reduce intestinal motility in clinical investigations and examinations [49, 50]. Glucagon reduces the tone of smooth muscle cells, leading to dilatation of large vessels and relaxation of the gastrointestinal tract [51, 52]. Given that glucagon relaxes smooth muscle cells, we hypothesized that glucagon also relaxes smooth muscle cells in small arteries and capillaries, resulting in vasodilation. Accordingly, we believe that glucagon thereby promotes its own SC absorption, which is supported by our research results, indicating that the pharmacodynamics of IP and SC-delivered glucagon are similar [26, 27]. The difference in size of insulin and glucagon molecules might also be a part of the explanation. In addition, insulin formulations consist of an equilibrium of insulin monomers, dimers, and hexamers. Because insulin monomers and dimers are small, they are rapidly absorbed from the SC tissue; however, the hexamers are too large to diffuse into the capillaries and must first dissolve to dimers and monomers before the molecules can be absorbed. Glucagon, on the other hand, is not arranged in oligomers when injected.

**Fig. 2:**
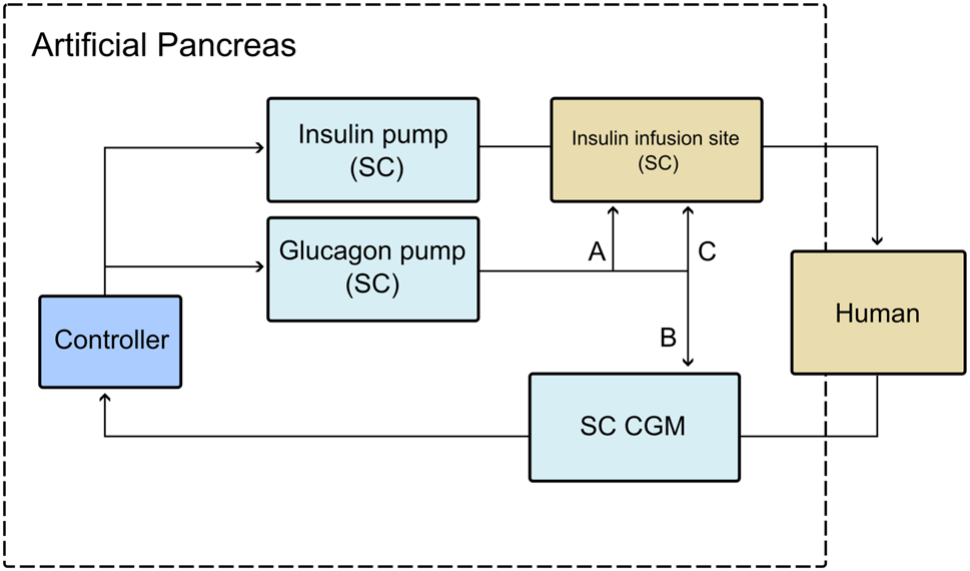
Illustration of a subcutaneous (SC) artificial pancreas with possible uses of glucagon to achieve a fully closed-loop system; **A** glucagon is used to prevent or treat hypoglycemia, **B** glucagon micro-boluses are used to enhance continuous glucose monitoring (CGM) performance by increasing local SC blood flow, **C** glucagon micro-boluses are used to accelerate absorption of insulin by increasing local SC blood flow

To explore the above-mentioned hypothesis, we recently performed a study in 22 healthy subjects and observed that SC micro-boluses of glucagon (0.1 and 0.01 mg) significantly increased the local cutaneous blood flow on the abdomen evaluated by laser Doppler technology [30]. As previous research has shown, increasing local blood flow affects the speed of insulin absorption and CGM performance. Hence, we propose using glucagon micro-boluses to enhance the performance of the AP system (Fig. 2). A double SC bihormonal AP that also utilizes glucagon as a vasodilative agent can be configured in several ways. By combining the site of SC hormone delivery, glucagon can be used to not only treat and prevent predicted hypoglycemia, but glucagon micro-boluses can also be used to increase local SC blood flow and thereby enhance the absorption of meal boluses of insulin and improve the postprandial dynamics of insulin absorption and improve the performance in future AP systems [30, 53]. Recently, we observed in a pig model that 0.1 mg og glucagon injected at the same site as SC insulin (NovoRapid^®^) injection, enhanced the insulin absorption (paper submitted).

Integrating hormone delivery and CGM has already been proposed to improve the robustness and practicality of the AP components. A study in 12 adults with DM1 was conducted in which the effect of glucagon micro-boluses was investigated both close to and remote from the CGM sensor. The study showed that glucagon injected 2 cm from the CGM did not significantly affect sensor accuracy [54]. However, micro-boluses of glucagon would most likely have to be administered closer to the CGM’s sensing element to enhance the performance of the CGM as the SC vasodilative effect of glucagon is very local and not detectable 15 mm from the injection site (“Personal communication; [Erlend Y. Munkerud and Mathilde H. Berge], 30 Jun 2021”).

The user friendliness of APs should also be considered. Going from a single-hormone SC AP to a conventional dual hormone SC AP entails a more complicated wearable system. Two hormone delivery lines with individual infusion sites are needed. With our proposed new, innovative, and disruptive use of glucagon in an SC bihormonal AP, only one infusion site and one two lumen infusion line would be needed. Thus, essentially only the size and weight of the infusion pump will be increased compared to present single-hormone hybrid SC APs.

Certain challenges will have to be addressed to develop and commercialize an AP system that employs glucagon micro-boluses to overcome the slow dynamics in the system. One challenge is the instability of liquid glucagon solutions. Glucagon is mainly prescribed as an emergency treatment of severe hypoglycemia and must be reconstituted before administration; however, it has been used for up to 24 h in clinical trials [21, 22, 24]. Glucagon easily forms amyloid fibrils over time when mixed in an aqueous solution; however, glucagon solutions and glucagon analogue solutions stable for several weeks at room temperature have been developed recently [55, 56], which will facilitate the use of glucagon in an AP [57]. The vasodilative properties of these stable glucagon formulations must be verified in patients with DM1, and the optimal dosage of glucagon to achieve the desired effect, i.e., the least effective dose to induce local SC vasodilation, must also be identified. The potential adverse effects of micro-dosed SC glucagon, both systemic and local, must be investigated. However, we have data from experiments on ourselves, indicating that much smaller doses of glucagon then 0.01 mg induce local SC vasodilation in healthy subjects. In addition, optimal algorithms must also be developed. However, it should be possible to accomplish all these tasks within a reasonable timeframe.

One can argue that even small amounts of SC-delivered glucagon may increase glucose levels, as is shown in studies with bihormonal AP systems [21]. However, the timing of glucagon delivery in relation to insulin levels is of importance as it has been shown that the efficacy of glucagon is related to insulin levels. Studies show that micro-boluses of glucagon correct hypoglycemia when insulin levels are low but often fail to influence on glucose levels when insulin levels are high [58, 59]. Using glucagon micro-boluses to enhance insulin absorption probably has a low potential for adverse effects on glucose levels as these will be given simultaneously as meal-time insulin boluses. On the other hand, if micro-boluses of glucagon are used to improve CGM performance, these boluses will have to be administered at regular intervals, at least during most of the daytime. It might include periods before meals, i.e., periods with low circulating insulin levels, low hepatic insulin effect, and low or non-measurable endogenous glucagon levels. Thus, using glucagon micro-boluses to improve CGM performance may increase glucose levels both due to the total delivered amount and the fact that these boluses will be administered during periods of low insulin levels. This effect might counteract the possible benefit of improvement in SC CGM, and consequently provide no benefit for the overall AP system performance.

When evaluating the possible effect of glucagon micro-boluses on blood glucose levels, it is also important to note that oral glucose intake stimulates glucagon release within minutes. This has been shown in healthy people, pancreatectomized subjects, and in patients with DM1 and diabetes mellitus type 2 [60–62]. Studies report that in response to high blood glucose levels, cells in the intestinal wall secrete glucagon [63]. This finding is further supported by the fact that treatment of DM1 patients with a long-acting formulation of a somatostatin analogue inhibits postprandial glucagon and reduces glucose excursions [64].

If micro-boluses of glucagon are used to enhance insulin absorption in a fully automated SC AP system, glucagon will mainly be administered when a meal has been confirmed and in concert with insulin meal boluses. In this scenario the postprandial release of glucagon has been initiated and the liver has already been saturated with glucagon from the intestine. In view of our data from pig experiments, indicating a hepatic first-pass effect of both insulin and glucagon [29, 65], the theoretical contribution from these SC glucagon micro-boluses to enhance insulin absorption is probably negligible compared to the gastrointestinal glucagon secretion after meal intake [62, 66]. On the other hand, the effect of these micro-boluses on SC insulin absorption because of increased local SC blood flow, as indicated by our recent data from healthy subjects [30], will probably be substantial. It is also supported by our resent experiments in pigs indicating faster insulin absorption (paper submitted).

## Discussion

At present, it appears that a single-hormone SC AP cannot achieve desired glucose targets for patients with DM1 because of the intrinsic slow dynamics in glucose sensing and insulin effect. This paper proposes two novel solutions to overcome these limitations in the AP system, i.e., an IP AP and a bihormonal SC AP that utilizes the recently discovered vasodilatory properties of SC glucagon micro-boluses.

Delivering insulin in the IP space is a physiologically reasonable solution for an AP. The pharmacokinetics resemble normal human physiology, with a high concentration hitting the liver before entering the systemic circulation. However, IP glucose sensing and glucagon delivery does not seem to be more appropriate that the SC solution. Thus, the improved performance of an IP AP seems to depend on the accelerated absorption of insulin and reduced systemic exposure to insulin. The IP approach is undoubtedly more invasive and, therefore, less acceptable for patients than an SC approach. Additionally, IP insulin delivery is hampered by several challenges, such as occlusion of the insulin delivery line, which has been reported in several trials of IP insulin delivery and infection at the site of the abdominal port [67]. In summary, we hold that this solution for an AP would be acceptable for a significant share of patients with DM1 only if glucose control approaches the non-diabetic range, i.e., close to 100% TIR.

An SC bihormonal approach is, however, far less invasive than the IP approach and is probably a preferred solution that can be used by most patients. Present research supports that double SC bihormonal AP improves glucose control compared to single-hormone AP. However, the system cannot achieve perfect glucose control. The recently identified vasodilatory effect of glucagon opens a new innovative application of glucagon in AP systems that may significantly impact AP research and development. Our results suggest that micro-boluses of glucagon increase local SC blood flow and might reduce the absorption time of meal boluses of insulin if the drugs are administered together. Combining the SC site for glucagon and insulin delivery would add minimal complexity to the system if stable glucagon formulations or analogues can be used. Additionally, this approach can utilize currently available pumps, whereas only the equipment for transcutaneous passage of insulin and glucagon and the controller algorithm will have to be developed.

Although the technological developments in diabetes management aim to improve the quality of life for diabetic patients, present modern technologies require user involvement in terms of time spent interacting with the technical devices and understanding the devices’ practical implications. Thus, the burden and potential adverse effects of using innovative technologies and treatment options for patients with DM1 must be weighed against the possible improvement in overall glucose control and, thus, reduced frequency of diabetic complications and increased quality of life. Even so, we hold that focus should turn to the potential of enhancing the absorption of insulin from the SC tissue by utilizing the vasodilative properties of glucagon and, concomitantly, using glucagon to treat and prevent hypoglycemia.

In conclusion, the aim of a fully automated AP is still not realized even after half a century of research efforts. Mimicking the function of a complicated regulatory process is not easy, and clearly, the most straightforward AP approach, the single-hormone double SC approach, is not going to succeed in achieving optimal glucose control. Different ways of improving the dynamics in the closed loop have been investigated, but no AP system, available or under development to date, holds the promise of an ideal AP with optimal glucose control and freedom from diabetes management. However, if glucagon can be used not only to prevent and treat hypoglycaemia but primarily to improve the performance of SC CGM and SC absorption of insulin, this new, innovative and disruptive use of glucagon may fulfil the dream of a true, fully automated AP suitable for most patients with DM1.

## Data Availability

This review article does not present new data.

## Declarations

### Patient and public involvement

The research group Artificial Pancreas Trondheim (APT) have consulted with the user board consisting of people with DM1. The user board has guided and provided feedback to the research conducted by APT.
